# The role of minerals in prebiotic evolution: Implications for identifying worlds with the potential for life’s origin

**DOI:** 10.1016/j.fmre.2026.07.002

**Published:** 2026-07-04

**Authors:** Houze Lu, Yan Li, Dehai Liang

**Affiliations:** a SKLab-DeepMinE, MOEKLab-OBCE, School of Earth and Space Sciences, Peking University, Beijing 100871, China; b Beijing Key Laboratory of Mineral Environmental Function, School of Earth and Space Sciences, Peking University, Beijing 100871, China; c Beijing National Laboratory for Molecular Sciences, Department of Polymer Science and Engineering and the Key Laboratory of Polymer Chemistry and Physics of the Ministry of Education, College of Chemistry and Molecular Engineering, Peking University, Beijing 100871, China

**Keywords:** Minerals, Origin of life, Prebiotic evolution, Habitability, Mineralogical indicators

## Abstract

Understanding the origin of life requires explaining how abiotic molecules transitioned into the first living systems within plausible geological settings. Increasing evidence suggests minerals as active participants in prebiotic evolution. By adsorbing and concentrating reactants, catalyzing key reactions, stabilizing intermediates, and providing protective microenvironments, minerals may have helped to overcome thermodynamic and kinetic barriers, thereby enabling a cascade of prebiotic reactions to proceed toward the earliest life. In this review, we synthesize current experimental and theoretical advances to examine the contributions of minerals across the successive stages of prebiotic chemistry, from the formation of simple precursors and biomolecular monomers to polymerization, compartmentalization, and protocell assembly. We propose that these processes were unlikely to have unfolded within a single environment because different prebiotic reactions imposed distinct physicochemical demands. Instead, prebiotic evolution probably proceeded through complementary reactions distributed among multiple geological settings, including hydrothermal vents, terrestrial hot springs, and potentially extraterrestrial niches, with intermediates being preserved and then transported across spatial and temporal geological scales to link otherwise disconnected evolutionary stages. Building on this view, we identify specific minerals capable of facilitating key reactions at different stages of prebiotic evolution and propose that their combined occurrence as characteristic mineral assemblages may provide mineralogical indicators for assessing a planet’s geological potential to support life’s emergence. This mineral-centered perspective complements conventional biosignature-based approaches in astrobiology, and highlights mineralogy as a powerful tool in the ongoing search for habitable worlds and life beyond Earth.

## 1 Introduction

The search for habitable worlds and detectable biosignatures beyond Earth is a central goal of planetary science. However, this effort remains fundamentally constrained by our incomplete understanding of how life first emerged and persisted on the early Earth. Currently, efforts to define the conditions under which life can arise mainly rely on reconstructing the physical and chemical environments of the prebiotic Earth. Minerals, as fundamental constituents of planetary crusts and surfaces, have increasingly been recognized as essential and active participants in prebiotic chemistry, driving transformations of energy and matter that may have enabled the origin and earliest evolution of life [[1], [2], [3], [4], [5]]. Although the specific timing of life’s first emergence remains unresolved, geological evidence shows that over 420 mineral species were already present during the Hadean Eon (>4.0 Ga) [6,7]. This unexpectedly high mineral diversity on prebiotic Earth has stimulated growing interest in the specific roles that minerals may have played in chemical evolution and whether some minerals actively contributed to life’s emergence.

Multiple hypotheses have been proposed to explain how life emerged through a series of distinct stages [8]. Among the earliest, the Oparin-Haldane hypothesis (also known as the primordial soup model), proposed that organic molecules formed from abiogenic precursors under the reducing conditions of early Earth [9,10]. This idea gained support from the famous Miller-Urey experiment, which demonstrated the abiotic synthesis of amino acids by spark discharge in a simulated primitive atmosphere [11]. Subsequent studies have further demonstrated that minerals may have facilitated these prebiotic processes. For example, elemental sulfur, transition-metal sulfides and oxides could facilitate the reduction of inorganic carbon or nitrogen species through photochemical or thermochemical redox reactions under mild prebiotic conditions, enabling sustained synthesis of simple organic building blocks [2,4,12,13]. Moreover, clay minerals have been shown to catalyze the polymerization of amino acids [14]. Another prominent theory, the RNA World hypothesis, initially proposed in the late 1960s [[15], [16], [17]] and later termed by Walter [18], suggests the first self-replicating functional polymer on the early Earth was RNA, which eventually evolved into the modern DNA-RNA-protein system seen today [19]. In particular, minerals also feature critically in this model: borate minerals, for example, can stabilize ribose and promote the assembly of pre-RNA molecules [20]. Collectively, these studies underscore the diverse and potentially indispensable roles of minerals in key prebiotic chemical processes, from small-molecule synthesis and molecular concentration to polymerization and the stabilization of genetic precursors.

Despite this growing recognition, most origin-of-life models and experimental systems remain focused on individual reactions or isolated stages, rather than on integrated reaction cascades or chemical networks operating within geologically plausible settings. Yet the emergence of life was almost certainly a progressive and multistage process, involving a sustained increase in chemical complexity from atoms and simple organic molecules to molecular building blocks, functional polymers, and eventually the first cellular life forms [9,10,18,21,22]. Validating a unified scenario to bridge the full transition from abiotic chemistry to life across multiple evolving stages represents a central challenge. Addressing this problem requires not only demonstrating individual catalytic effects of minerals, but also evaluating whether specific mineral assemblages, coupled with specific redox conditions, fluid compositions, energy sources and environmental dynamics, could have enabled linked prebiotic reaction cascades over extended timescales. Such a framework is essential for connecting laboratory prebiotic chemistry to planetary-scale scenarios of life’s emergence.

This perspective is equally important for astrobiology. The direct detection of extraterrestrial life is limited by the scarcity of accessible samples from Mars or other planetary bodies. Consequently, current approaches rely largely on the remote sensing of putative biosignatures [23], which are often unstable and difficult to interpret unambiguously. Mineralogical signatures provide a potentially more robust, stable, and observationally accessible alternative. Studies over the past few decades have shown that different minerals can perform diverse prebiotic functions [[1], [2], [3],^24^,^25^]: they may act as catalysts or reactants, concentrate organic compounds through adsorption, or shield biomolecules from UV radiation and other harsh conditions. These functions suggest that mineral assemblages may record not only whether an environment was broadly habitable, but also whether it possessed the capacities necessary to drive prebiotic evolution. Therefore, the integration of mineralogical diagnostics, especially those minerals indicative of enabling specific prebiotic reactions, into detection strategies promises to refine the interpretation of planetary observations in the search for life beyond Earth.

In this review, we summarize current understanding of the multifaceted roles that minerals played across various stages of prebiotic evolution, highlighting their important and distinct contributions to the chemical evolution toward life. We compare and evaluate major origin-of-life hypotheses associated with different geological settings, with particular emphasis on how specific mineral assemblages may have facilitated and sustained key reaction cascades necessary for the emergence of life. On this basis, we propose a set of diagnostic mineralogical signatures that may indicate different stages of prebiotic reactivity in planetary environments. These mineral-based proxies may offer a useful framework for assessing habitability and reconstructing plausible prebiotic pathways. By integrating these mineral-focused perspectives, this review aims to complement and enhance existing frameworks for detecting habitable worlds and to strengthen broader efforts to understand the origins of life.

## 2 Roles of minerals in prebiotic evolution

### 2.1 Synthesis of organic building blocks

#### 2.1.1 Organic precursors

The formation of simple organic precursors represents a crucial first step in prebiotic evolution. While the origin of these foundational molecules remains an open question, mineral catalysis, particularly by metal sulfides, has been proposed as a key mechanism for their generation. Among the most influential theories, the iron-sulfur world hypothesis emphasizes the catalytic role of metal sulfides in hydrothermal systems, where they could reduce inorganic carbon into small organic compounds such as formate, acetate and methanethiol [21,26]. Under simulated hydrothermal conditions (up to 100 °C, ambient pressure), coprecipitated FeS-NiS facilitate the carbonylation of carbon monoxide (CO) and methanethiol (CH_3_SH) to produce acetic acid (CH_3_COOH) [2]. This reaction is proposed to occur via a surface-bound organometallic mechanism, where coordinatively unsaturated metal sites (e.g., Ni^2+^, Fe^2+^) on the sulfide surface adsorb and activate the reactants [2]. At higher temperatures and pressures (250 °C, 50–200 MPa), FeS promotes the formation of organometallic phases from CO and alkyl thiols, indicating a potential pathway toward more complex carbon-bearing intermediates and more advanced biomolecular building blocks [24]. The potential prebiotic importance of metal sulfides is further supported by the conserved occurrence of transition metal-S motifs as catalytic centers in modern metalloproteins [27], reinforcing the hypothesis that ancient mineral-mediated reactivity may have been inherited by early biochemical systems.

Nitrogen fixation is another parallel challenge in prebiotic synthesis, as the reduced nitrogen species (e.g., NH_3_, NH_4_^+^) are required for the formation of amino acids and nucleotides. While a reducing atmosphere rich in methane (CH_4_), hydrogen (H_2_) and ammonia (NH_3_), could have generated ammonia and hydrogen cyanide (HCN) directly [28,29], some models now favor a less reducing, namely neutral to mildly oxidizing early atmosphere where nitrogen existed primarily as inert N_2_ [30,31]. In such scenarios, lightning and photochemical reactions would have produced oxidized nitrogen species, such as nitric oxide (NO), nitrate (NO_3_^−^) and nitrite (NO_2_^−^) [32], which required further reduction to NH_3_ or NH_4_^+^ before entering prebiotic synthesis. Here again, minerals, especially metal sulfides, could have provided a viable solution for the reduction. Evidence shows that metal sulfides can serve as effective reductants across a wide range of prebiotically plausible settings. For example, commercial FeS reduces nitrate and nitrite to NH_4_^+^ at room temperature, especially in acidic environments [33]. Under simulated hydrothermal conditions (300 °C, 50–500 MPa), natural metal sulfides such as pyrrhotite (Fe_1-x_S), chalcopyrite (CuFeS_2_), millerite (NiS) and chalcocite (Cu_2_S) readily convert NO_3_^−^ and NO_2_^−^ to NH_4_^+^, with pyrrhotite achieving complete reduction of nitrate and 46% of nitrite within 90 min [34]. Even under milder conditions (≤120 °C), pyrite(FeS_2_) can facilitate the reduction of NO_2_^−^ and NO_3_^−^ to NH_4_^+^ [35], while FeS operates effectively over a broad pH range at room temperature, using sulfur vacancies as reactive sites [33,36]. Collectively, these findings suggest that mineral-driven nitrogen reduction was not restricted to high-temperature hydrothermal systems, but may also have occurred in cooler, more widespread environments. Such reactivity would have broadened the environmental window for prebiotic nitrogen fixation and lowered the energy barriers to the accumulation of reduced nitrogen.

Moreover, mineral-mediated nitrogen reduction may have extended beyond terrestrial settings. Fe, Ni and their alloys (e.g., awaruite Ni_80_Fe_20_ and tetrataenite Ni_50_Fe_50_) have been shown to reduce NO_2_^−^, NO_3_^−^ and even N_2_ to NH_4_^+^ at 200 °C, indicating that meteorite-delivered minerals may have provided an additional extraterrestrial source of reduced nitrogen [37]. All these studies indicate the versatile and critical contribution of mineral catalysts in supplying the essential molecular precursors for life’s emergence.

#### 2.1.2 Key biomolecular monomers

Beyond the formation of simple organic precursors, prebiotic evolution required the synthesis, stabilization and accumulation of essential biomolecular monomers, such as amino acids, nucleobases, and sugars. Increasing evidence indicates that minerals contributed to each of these steps by catalyzing reactions, modulating product selectivity and protecting labile intermediates under plausible early Earth conditions.

**Amino acid synthesis.** Hydrothermal systems have long been regarded as plausible environments for the prebiotic synthesis of amino acids. Under temperatures of 80–120 °C, Ni/Fe sulfides and hydroxides can catalyze the conversion of inorganic molecules like CO and CN^−^ into ɑ-keto acids, which are subsequently aminated to generate amino acids [12,38]. Partially oxidized iron phases, such as Fe(OH)_2_, exhibit catalytic activity due to the redox reactivity of surface Fe^2+^ sites. In these systems, amino acid yields are favored by higher Fe(II)/Fe(III) ratios, elevated temperatures and pH [38,39]. Besides sulfide minerals, Fe-rich silicate clays, such as saponite (formed through serpentinization), can promote the synthesis of aromatic amino acids. At 100–150 °C, Fe^3+^ sites on saponite surfaces act as Lewis acid sites to accept electrons, catalyzing the alkylation and amination of pyruvate to generate tryptophan and related compounds [40]. Together, these findings suggest that diverse mineral phases could have expanded both the environmental range and chemical scope of prebiotic amino acid synthesis.

**Nucleobases formation.** Minerals can facilitate the formation of nucleobases, often starting from formamide as a key precursor [41]. Under mild thermal conditions (∼160 °C), silica (SiO_2_), alumina (Al_2_O_3_), calcium carbonate (CaCO_3_) and Y-type zeolite can convert formamide into nucleobases such as cytosine and adenine [42]. Transition metal oxides like titanium dioxide (TiO_2_) can catalyze the synthesis of adenine and thymine under 160 °C [43]. It has also been demonstrated that UV-irradiated TiO_2_ (001) surfaces promote the formation of all five canonical nucleobases via photocatalytic pathways [13]. Clay minerals such as montmorillonite can promote the thermal condensation of formamide to yield uracil, cytosine, and adenine, while also protecting the products from degradation [44]. In addition, other minerals such as borates, phosphates, iron sulfides, and zirconia, exhibit catalytic potential under thermal conditions, which supports a general model of mineral-enabled, thermally driven nucleobase formation in hydrothermal settings [41].

**Ribose synthesis and stabilization.** Sugars represent a challenging class of prebiotic products because they are readily degraded under many plausible environmental conditions. Ribose, a type of monosaccharide (pentose) and essential building block for nucleic-acid backbones, can be generated abiotically through the formose reaction, in which formaldehyde is converted into sugars [45]. However, this reaction is typically non-selective and prone to producing unstable mixtures. Borate minerals have been proposed as a crucial means of imposing chemical control on this system. By complexing with intermediates such as glyceraldehyde and glycolaldehyde, borates can steer the reaction toward pentose formation, including ribose [46]. Notably, borate also stabilizes ribose itself by forming relatively unreactive complexes with its cyclic structure to inhibit degradation [3]. Thus, borate minerals were important for the formation and the accumulation of ribose, both of which would have been essential for subsequent nucleotide-forming reactions.

Considering the strong chemical affinity between ribose and borate, high borate concentration may enrich ribose, and drive the subsequent prebiotic synthesis processes. Thus, the availability of borate minerals on Earth during the Hadean era is the key to assessing the plausibility of this scenario. Although the earliest borate minerals discovered hitherto are tourmaline dating to around 3.8 Ga [47,48], possible existence of borate minerals before 4.0 Ga cannot be excluded. Several geological mechanisms have been proposed for early boron enrichment, including weathering of exposed granitic continental crust and leaching of hydrothermal fluids from oceanic crust into oceans and shallow water bodies [20,49]. Another proto-arc model proposed for the tectonic evolution during 3.8 to 3.7 Ga invokes the extraction of boron by deep fluids from felsic igneous rocks into the ocean [50]. This model may also explain the enrichment of boron before 4.0 Ga, if similar geological scenarios did exist during Hadean [49,50].

**Extraterrestrial organics and mineral protection.** The contribution of minerals to the inventory of key biomolecular monomers may have extended beyond strictly terrestrial environments. Extraterrestrial delivery has long been considered an additional source of prebiotic organic matter, especially during episodes of intense bombardment [51]. As one of the most primitive meteorites, carbonaceous chondrites, such as the CM2 chondrite Murchison meteorite, host over 80 amino acids, nucleobases, and ribose [52]. Interplanetary dust particles (IDPs) may likewise have provided a steady flux of essential elements such as C, N and P to early Earth [53]. While the formation mechanisms of these compounds in parent bodies remain unclear, proposed pathways include photochemical or thermal reactions of precursors such as hexamethylenetetramine (HMT) [54], or aqueous alteration within icy planetesimals [55]. In this context, minerals likely played a vital protective role during transport and atmospheric entry, shielding organics from UV radiation and thermal degradation. Coordination complexes between organic molecules and mineral surfaces, such as iron cyanocarbonyl compounds detected in meteorites, may have ensured the stable delivery of key precursors like cyanide to early Earth [56]. Extraterrestrial minerals and mineral-organic assemblages may therefore have complemented endogenous geochemical processes by supplying and preserving a broader inventory of prebiotic monomers.

### 2.2 From monomers to polymers

#### 2.2.1 The role of minerals in the transition from monomers to functional polymers

Beyond facilitating the synthesis of biomolecular monomers, minerals likely played a decisive role in driving their assembly into oligomers and functional polymers, which is a critical transition toward biological complexity. Through adsorption, catalytic activation, and structural templating, mineral surfaces enabled key polymerization reactions under prebiotically plausible conditions.

**Concentration and activation of monomers.** For polymerization to occur, monomers must first be concentrated and chemically activated. Mineral surfaces can adsorb and condense a wide range of organic molecules, such as amino acids, nucleobases, nucleotides, and sugars, through electrostatic and coordination interactions [57,58]. The efficiency of adsorption depends on multiple environmental variables, including solution composition, ionic strength, pH, surface area, temperature, and other factors [57,58]. Amino acids, which typically possess both carboxylic acid and amine groups, often exist as zwitterions and can be adsorbed onto mineral surfaces even under neutral conditions [59]. Such adsorption facilitates localized enrichment, which not only increases molecular density but can also pre-organize molecules into configurations favorable for reaction.

**Catalysis for peptide formation.** The abiotic formation of peptides presents a particularly difficult problem because peptide condensation is thermodynamically unfavorable in dilute aqueous environments such as ancient oceans [14,60]. Minerals may have helped overcome this constraint by catalyzing peptide bond formation and, under some conditions, coupling condensation to environmental cycles that repeatedly drive dehydration and rehydration. Common mineral phases such as pyrite, calcite and rutile have been shown to catalyze both peptide bond formation and peptide hydrolysis without changing the overall reaction equilibrium [14]. Under mild heating or wet-dry cycling conditions, clay minerals (e.g., kaolinite, bentonite, montmorillonite) can accelerate glycine oligomerization and the formation of mixed di- and tripeptides [50,61]. In laboratory simulations of hydrothermal settings, metal sulfides such as (Fe,Ni)S facilitate peptide synthesis from glycine, phenylalanine, and tyrosine at 100 °C, in the presence of CO and H_2_S (or CH_3_SH) [62]. According to the “iron-sulfur world” hypothesis, pyrite has been proposed to catalyze the oligomerization of amino acids at aqueous-mineral interfaces. This reaction can be achieved under extreme hydrothermal conditions (∼500 K, ∼20 MPa) with carbonyl sulfide (COS) present. Theoretical studies further suggest that pyrite surfaces may lower activation energy for peptide condensation under such conditions [63]. These findings suggest that mineral surfaces may have facilitated peptide synthesis by creating localized physicochemical settings in which condensation became more competitive with hydrolysis.

**Chirality selection and amplification.** The emergence of homochirality represents another major transition between simple prebiotic chemistry and functional biopolymers. Although chirality is widespread in abiotic systems, living organisms overwhelmingly use only one enantiomeric form in core biological molecules (e.g., the l-amino acids in proteins and d-configured nucleotides in DNA and RNA). How this symmetry breaking has arisen remains unresolved, but minerals may have contributed both to the initial selection of one enantiomer and to the subsequent amplification of small chiral excesses. Naturally occurring minerals can display chirality in several forms. According to Lee et al., these minerals can be categorized into three types: enantiomorphic minerals composed of intrinsically chiral unit cells (e.g., α-Quartz); achiral minerals with chiral faces (e.g., calcite, hematite); and minerals with local chiral sites associated with crystal imperfections or structural defects (e.g., clay minerals) [64]. These minerals may contribute to chiral selection through enantioselective adsorption of enantiomers. For example, Hazen et al. first reported the enantioselective adsorption of aspartate on calcite surfaces [65]. Such adsorption may subsequently facilitate mineral-organic interactions on mineral surfaces or interfaces such as producing certain enantiomers or homochiral polymers and preservation of enantiomers [64,66]. However, the efficiency of enantiomeric selectivity via the mineral adsorption was generally low [64], suggesting additional mechanisms of chiral amplification were required. Certain autocatalytic reactions yielding high enantiomeric excess may provide a solution. The Soai reaction, for example, can amplify extremely small initial chiral biases to produce near-enantiopure products, and minerals such as quartz can act as chiral triggers in this process [67,68]. The chiral amplification may also be achieved by the physicochemical coupling in the environment, as recent studies showed that the combination of wet-dry cycling environments and mineral adsorption can remarkably improve the enantiomeric excess of several peptides [69]. Furthermore, different types of minerals possessing chirality created ubiquitous chiral crystal environments on early Earth, which may also have promoted the chiral amplification toward biological homochirality [64,66].

**Templating and catalysis in nucleotide oligomerization.** A central question of the origin of genetic polymers is how nucleotide monomers became assembled into oligomers that stored information and, ultimately, supported heredity and catalysis. In most origin-of-life models, RNA is considered to have preceded DNA because it combines informational and catalytic properties, thereby providing a plausible molecular bridge before the establishment of the modern DNA-RNA-protein system [70]. Consequently, experimental studies of mineral-mediated nucleotide polymerization have focused primarily on ribonucleotides. Among the minerals investigated, montmorillonite is the most extensively studied candidate for RNA oligomerization [[71], [72], [73], [74]]. This clay consists of tetrahedral-octahedral-tetrahedral layered sheets in which isomorphic substitutions (e.g., substitution of Al^3+^ for Si^4+^ in tetrahedral sheet, Mg^2+^ for Al^3+^ in octahedral sheet) create charge deficiencies and reactive surface sites that can bind nucleotide monomers [72,75]. These features enable montmorillonite to efficiently concentrate nucleotides, enhance phosphodiester bond formation, and accelerate oligomerization relative to hydrolysis, in some systems by factors of 100–1000 [76]. Mineral surfaces therefore not only enhance reaction rates, but also shift the balance of competing pathways toward polymer growth.

During oligomerization, longer oligomers mean higher complexity and more genetic information can be stored. Thus, it is important to examine the ability of montmorillonite in extending the oligomers. In the presence of montmorillonite, oligomers with 10–20 monomer units can be generated from activated RNA monomers such as ImpA and ImpC in aqueous solution at pH 8.0 [77]. Longer oligomers can be obtained by modifying the reaction conditions and activating the phosphate group. Through “feeding” strategies with daily additions of activated monomers, RNA oligomers up to 40–50 units long were synthesized under prebiotically relevant conditions [73,78]. Furthermore, montmorillonite can promote sequence-specific and enantioselective polymerization, offering a plausible route to the first genetically informative polymers [72,75].

#### 2.2.2 Protective functions of minerals in organic molecules preservation and stabilization

The preservation of fragile biomolecules under the harsh early Earth conditions was as critical as their synthesis. Clay minerals function as effective molecular shelters due to a combination of key properties: high surface area for adsorption, small pore sizes that limit radiation penetration, and interlayer domains that isolate organics from the environment [25]. This protective role extends from amino acids to more complex molecules like nucleic acids and functional ribozymes, confirming their importance in prebiotic chemistry [78,79]. These findings highlight the role of minerals as molecular shelters in prebiotic environments. Notably, the presence of Fe^2+^ can severely influence molecular preservation by driving the oxidative degradation of organics via photo-Fenton reactions [80].

#### 2.2.3 Toward higher complexity: functional polymers, compartments and protocells

If the transition from monomers to polymers marked the emergence of chemical complexity, the next major step was the spatial organization of that chemistry into discrete compartments. Compartmentalization is widely regarded as a prerequisite for the origin of life because it enables the physical containment of reactive molecules, maintains local chemical integrity and creates microenvironments in which catalysis, molecular selection and replication can occur more efficiently. Prebiotic compartments may have helped segregate functional molecules from less productive reaction products, thereby favoring the persistence and accumulation of chemically advantageous systems [81,82].

Minerals (such as positively charged pyrite) can provide accumulation sites for amphiphilic lipids on their surfaces, and potentially support the subsequent membrane formation [21]. Clay minerals are reported to drive the prebiotic cellular compartmentalization. Specifically, montmorillonite can catalyze the formation of vesicles from fatty acids, and be encapsulated together with adsorbed RNA inside the vesicles [83], thereby achieving the co-formation of catalytic components and membrane compartments. Other mineral surfaces, including kaolinite, quartz, and pyrite, have been shown to direct the organic amphiphiles to form vesicles [84]. These findings suggest that mineral-assisted membrane assembly may have been a relatively widespread phenomenon on the early Earth rather than a process restricted to a single mineral class. More broadly, they support the view that minerals acted as catalysts and templates not only for molecular synthesis and polymerization, but also for the emergence of higher-order structures in which functional polymers and membrane compartments became physically coupled.

## 3 Overcoming constraints in mineral-mediated prebiotic synthesis

The prebiotic synthesis at different stages of chemical evolution is constrained by a set of critical environmental conditions such as available energy sources and suitable geological settings. Understanding these constraints is critical to comprehensively know the pattern of chemical evolution toward the earliest life on Earth.

### 3.1 Overcoming energy constraints

Sustained and substantial energy input was essential for powering prebiotic reactions. Early stages, including the reduction of inorganic carbon and nitrogen, required high-energy conditions that hydrothermal systems could plausibly have supplied [85]. Sulfide minerals are well-studied and assumed to have played a critical role due to their ability to convert diverse energy sources (e.g., heat, light, and chemistry) into electron energy, which is directly utilizable by reactants. Two principal mechanisms have been proposed. The first relies on deep-sea hydrothermal settings, where metal sulfides in black chimneys can efficiently convert thermal energy into electrical energy (i.e., electron flow), driving the synthesis of organic precursors [85] against thermodynamic barriers. The second mechanism involves the photoelectric effect of semiconducting minerals. Sulfides and natural sulfur (S⁰), which were abundant in early terrestrial hot springs, can absorb photons with energies higher than their bandgap, thereby exciting electrons from the valence band to the conduction band [4]. This photoexcitation endows the mineral with sufficient reducing power to convert inorganic carbon into organic molecules (Fig. 1). For example, sphalerite (ZnS) can reduce CO_2_ to formate (HCOOH) because its conduction band edge (−1.37 V vs. NHE, at pH 7.0) [86] is more negative than the redox potential of CO_2_/HCOOH (−0.61 V vs. NHE at pH 7.0) [87], thereby providing a thermodynamic driving force for photoinduced electron transfer [88]. Similarly, alabandite (MnS) reduces HCO_3_⁻ to formate, acetate and other organics via an analogous photocatalytic mechanism [89]. Notably, natural sulfur possesses the highest photoelectron energy among known semiconducting minerals, extending the range of reducible substrates under UV light [4,90,91], including overcoming the large negative potential of one-electron reduction CO_2_/CO_2_˙^−^ (−1.90 V vs. NHE, pH = 7) [92]. Once thermal or light energy has been converted into electron energy, redox-active metal sites on mineral surfaces (e.g., Fe^2+^) not only serve as effective adsorption sites for inorganic carbon but also mediate the transfer of electrons from the mineral to adsorbed molecules. In this way, minerals translate naturally available energy into chemical energy in the form of reducing equivalents, thereby overcoming the energetic bottlenecks that would otherwise have constrained the emergence of primordial life.

**Fig. 1:**
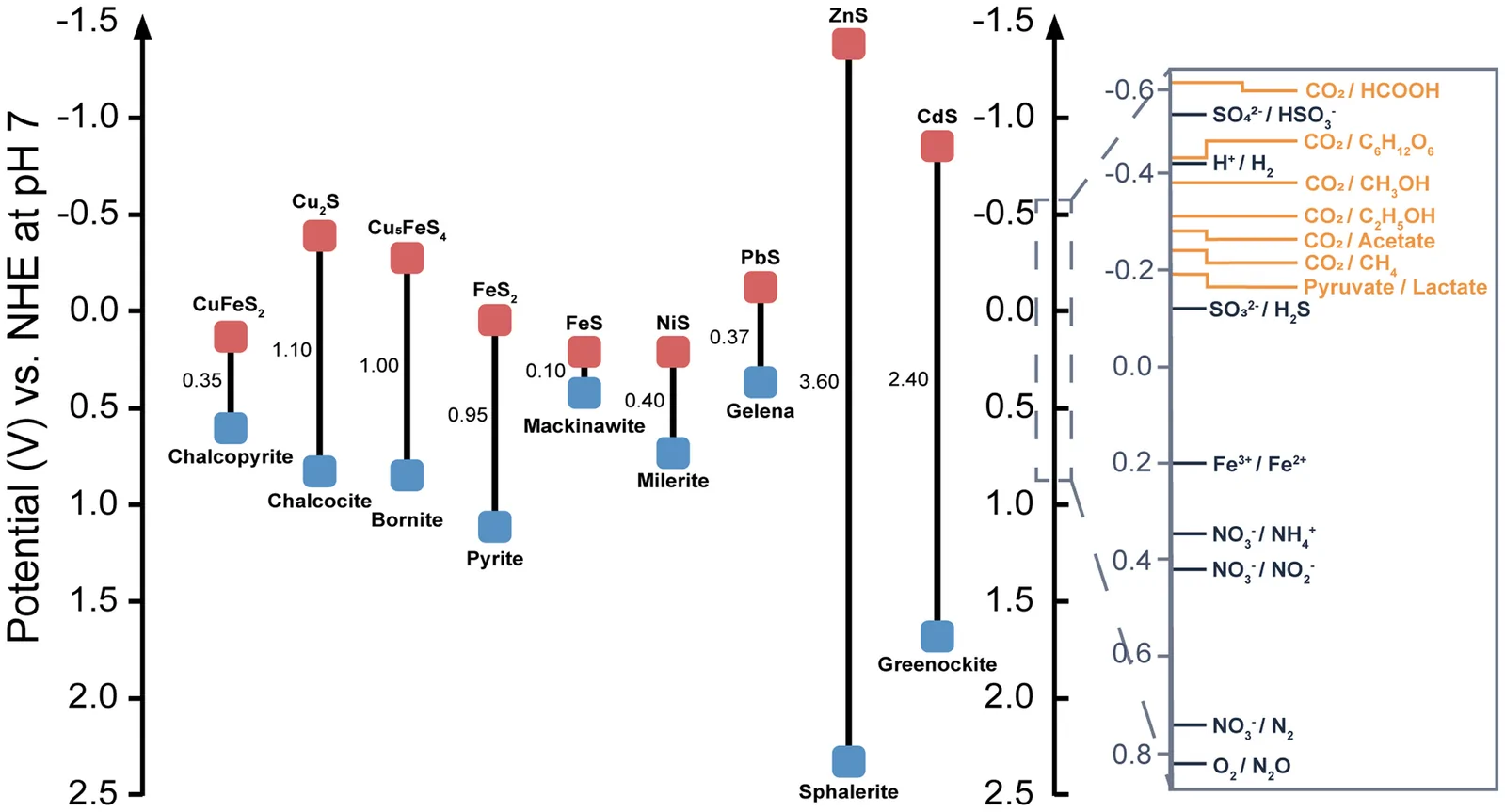
**Band-edge potentials of metal sulfides (vs. NHE, pH 7.0) and redox potentials for CO_2_ reduction and selected redox couples.** Mineral bandgap data are from [93]; reduction potential data are from [94].

### 3.2 Overcoming geological constraints

Beyond energetic limitations, prebiotic synthesis was also constrained by the fact that no single geological setting could simultaneously satisfy the diverse environmental requirements of all reaction stages. A wide range of environments, including Darwin’s “warm little pond”, primordial soup scenarios, and various hydrothermal systems, have been proposed as potential sites for life’s origin [9,10,21,26,95]. However, each of these settings provides only a subset of the physicochemical conditions that are favorable for prebiotic synthetic reaction [96]. Different stages of chemical evolution imposed distinct constraints on temperature, redox state, hydration, solute concentration, interfacial properties, and energy supply. As a result, environments favorable for one class of reactions may have been unsuitable for others. Given both the complexity of the prebiotic environment and the functional requirements of even the simplest protocellular systems, it is unlikely that the entire prebiotic pathway from abiotic molecules to early life unfolded within a single geological setting.

Minerals are central to overcoming this geological constraint because they provide functional continuity across otherwise disparate environments. Hydrothermal systems are widely regarded as ideal settings for the occurrence of organic precursors and simple molecular building blocks (Fig. 2a). These environments provide thermal energy and redox disequilibria that can drive key processes such as inorganic carbon reduction and carbon-chain elongation. Importantly, sulfide minerals in hydrothermal chimneys may have played active roles in this stage by facilitating electron transfer enabled by thermoelectric conversion under steep thermal gradients [85]. At the same time, however, many hydrothermal environments, especially high-temperature vent systems exceeding 300 °C, are poorly suited to later stages such as polymerization because elevated temperatures and hydrolysis-prone conditions tend to destabilize macromolecules and inhibit condensation reactions.

**Fig. 2:**
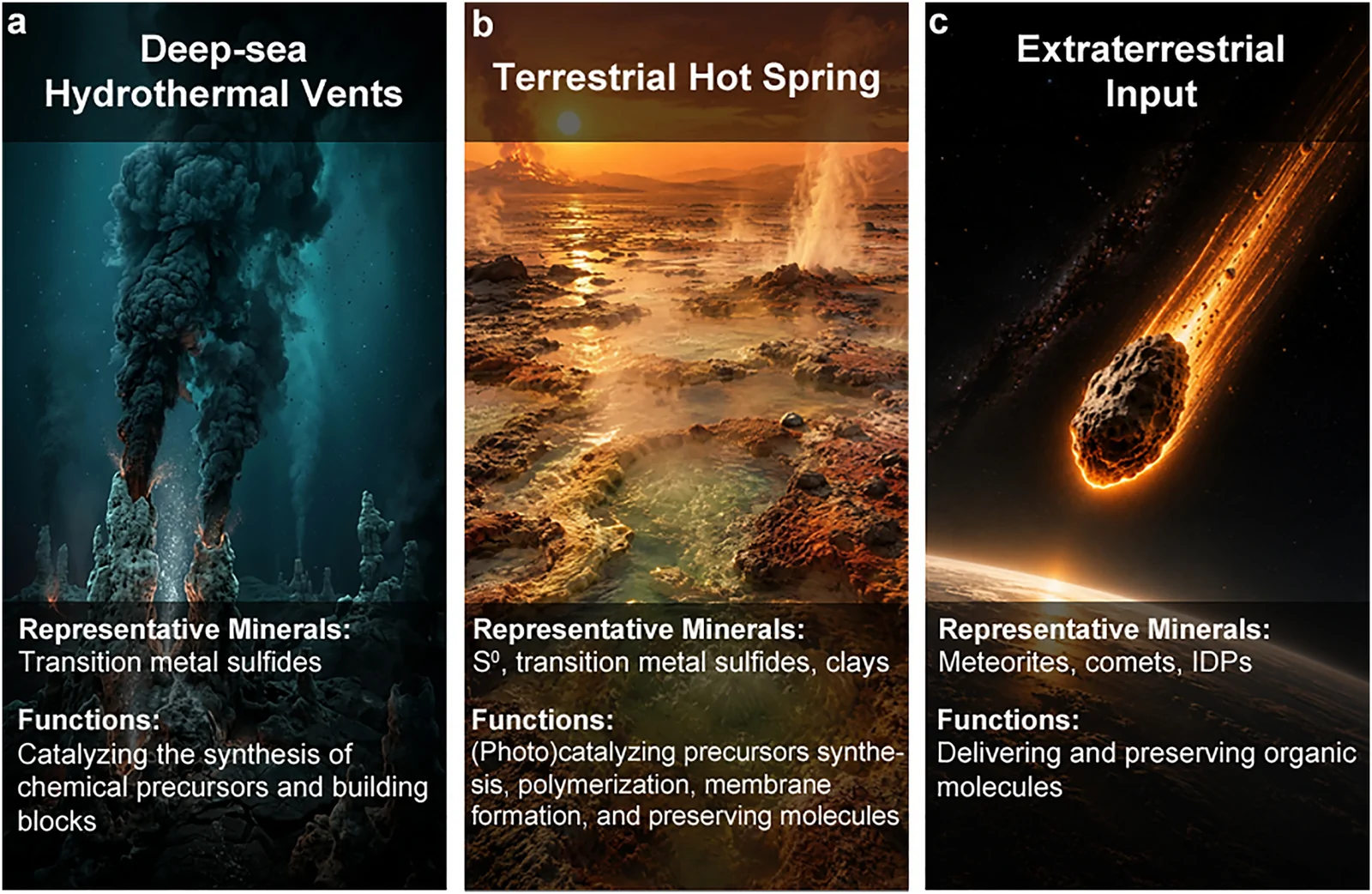
**Potential environments for the origin of life and the corresponding roles of minerals.** (a) In the deep-sea hydrothermal vent hypothesis, metal sulfides play a critical role in the formation of chemical precursors and building blocks. (b) In terrestrial hot spring, minerals may concentrate and stabilize molecular building blocks, catalyze polymerization under wet–dry cycling conditions, and provide protection against environmental stresses. (c) Extraterrestrial input of organic matter through meteorites, comets and IDPs. Minerals are the carriers and preservers of organic compounds and may possibly take part in the prebiotic synthesis of organic compounds in extraterrestrial environments. (Background images are modified based on AI-generated illustrations).

By contrast, the polymerization of building blocks and the assembly of functional polymers likely required fluctuating, non-equilibrium environments characterized by thermal gradients and changes in water activity and solute concentration. For instance, the formation of peptide bonds and phosphodiester bonds requires dehydration, and thus dry environments or wet–dry cycling regimes would favor reactant concentration and subsequent reactions. Here again, minerals may have helped overcome environmental limitations by concentrating reactants on their surfaces and lowering kinetic barriers to polymer formation. In particular, clay minerals have been reported to adsorb organic molecules and catalyze the formation of higher peptides under wet–dry cycling conditions, especially in the presence of salts [97]. Similar mineral surfaces may also have promoted nucleotide organization and template-directed chemistry. In the case of RNA replication, RNA oligomers may serve as templates. During replication, heating–cooling cycles could have played a crucial role: duplex structures would dissociate at the hot side, while template-directed synthesis of single strands could proceed at the cooler side [22]. Therefore, terrestrial hot springs offer promising sites for this stage because they combine heat and chemical energy, mineral surfaces, along with periodic wet–dry or heating–cooling cycles that could promote peptide formation and RNA oligomer growth [98]. In such settings, prebiotic polymer synthesis may have been strongly enhanced by mineral-assisted concentration and catalysis on exposed or intermittently wetted surfaces (Fig. 2b). Shallow-water alkaline hydrothermal systems may have offered similar advantages by combining both pH and redox gradient with periodic environmental fluctuations [99].

For compartmentalization and protocell emergence, suitable environments must support the aggregation of amphiphilic molecules and reactions within confined microspaces [81]. It is noteworthy that this process does not require continuous external energy input, but is strongly influenced by exterior physicochemical conditions such as ionic strength, pH, temperature, and surface charge. Minerals could have been especially important here by providing interfaces that stabilize amphiphilic assemblies and porous structures that localize reactants. Positively charged mineral surfaces, for example, may favor the stability of phospholipid vesicles, whereas mineral pores can create confined volumes that enhance molecular encounter rates and protect fragile intermediates. Alkaline hydrothermal vents may be a candidate for this stage. Their porous mineral structures, including FeS-rich precipitates, could have acted as primitive compartments, while natural pH and redox gradients across vent walls may have continued to drive prebiotic chemistry [100]. In such environments, organic molecules may have accumulated within mineral pores or on mineral surfaces, promoting membrane assembly and, ultimately, the emergence of protocell-like systems.

### 3.3 Linking successive stages of prebiotic synthesis

Within the framework of chemical evolution, the origin of life can be viewed not as a single event but as the outcome of an extensive network of interconnected prebiotic reactions. Over geological timescales, simple inorganic and organic precursors were progressively transformed into increasingly complex molecular systems, ultimately leading to the emergence of the earliest life. We therefore propose that this process is best understood as a cascade of successive prebiotic reactions. In this view, the early Earth functioned as a planetary-scale reaction system, in which minerals located in different environments acted as catalysts, adsorbents, templates, and protective scaffolds that facilitated distinct stages of prebiotic synthesis. Within localized environments, minerals can facilitate specific reactions, while the products of earlier stages had to be preserved, transported, and incorporated into later-stage processes in other environments. Through such sequential coupling, prebiotic chemistry could have advanced gradually from small molecules to larger polymers, from simple reaction systems to more integrated and life-like assemblies.

The timescale over which this transition occurred remains uncertain. However, the successive reactions leading to life were unlikely to occur in a strictly continuous sequence. A more plausible scenario is that some earlier reactions either persisted over extended periods or produced sufficient and relatively stable products that could exist on geological timescales. These products, once accumulated in adequate quantities, could then be transported to other new favorable environments for subsequent reactions. In this context, “successive” should not be interpreted as immediate or uninterrupted, but rather as a stepwise progression in which each stage created the material basis for the next.

This framework highlights two central requirements for the continuity of chemical evolution. The first is that prebiotic reactions must have overcome the energetic and material limitations of the early Earth. The second is that the reactants and products of one stage must have been coupled spatially and temporally to the reactions of later stages, allowing chemical complexity to accumulate rather than dissipate. Minerals are likely to have been critical to both requirements: they could lower energetic barriers, concentrate and stabilize intermediates, and provide persistent substrates linking otherwise separated stages of prebiotic synthesis.

### 3.4 Spatial and temporal coupling of successive prebiotic reactions

As discussed, the emergence of life can be understood as the outcome of a cascade of successive prebiotic reactions. This requires the synthesis of key molecular species as well as their progressive integration across broad spatial and temporal scales. Such continuity would have depended on transport processes and environmental evolutions that linked otherwise distinct stages of chemical evolution into a coherent prebiotic trajectory.

Central to this process was the spatial coupling of different geological settings, each hosting specific mineral assemblages that catalyzed critical yet distinct reactions. For example, deep-sea hydrothermal vents rich in metal sulfides could provide a highly reducing environment favorable for the synthesis of simple organic precursors and early building blocks. However, their high-temperature feature was unsuitable for the polymerization and stabilization of complex biopolymers. Instead, terrestrial hot springs [98] and shallow-sea hydrothermal vents [99], with lower temperature and more importantly, their fluctuating hydration environment, offer more favorable conditions for monomer condensation, oligonucleotide replication, and compartmentalization. In this way, different geological settings may have supported complementary stages of chemical evolution, with minerals in each environment facilitating the reactions most compatible with local conditions.

The spatial coupling of these settings may have been achieved through both local transport processes and long-distance redistribution. At local scales, hydrothermal fluids and seawater could establish continuous convection within porous sediments and chimney structures, thereby sustaining the input, mixing, and recirculation of chemical species, maintaining a dynamically stable reaction system [101]. Hydrothermal plumes may have dispersed dissolved organics and fine mineral particles into the surrounding waters, extending the chemical influence of vent systems beyond their immediate source regions. At larger scales, deep-ocean upwelling could transport products formed in deep-sea hydrothermal systems into shallower marine environments, where they may further accumulate in coastal sediments. In addition, geothermal fluid flow could transport the prebiotic organics while enriching them by forming a geo-microfluidic system through crack-like geo-compartments [102], thus linking the separated springs or hydrothermal systems. Coupling with the atmosphere may have provided an additional transport pathway: volcanic outgassing and aerosol formation could have facilitated the dispersal of reactive organic species, which were then returned to surface and ocean systems by rainfall. In addition, impact events may have ejected and redistributed both minerals and prebiotic organics across regional to planetary scales. Together, these processes provide plausible mechanisms by which spatially separated reaction settings could have been integrated into a broader prebiotic network.

Notably, the cascade leading to life does not need to be continuous in time either, provided that key intermediates could persist long enough to participate in later stages. On the one hand, springs/vents sites occurred in swarms may help these intermediates last longer [103], thus supporting the long-lasting occurrence of prebiotic reactions. On the other hand, many early syntheses may have occurred intermittently, with products accumulating during favorable episodes and persisting over geological timescales before being transported to settings conducive to the next stage. Minerals could have played an especially important role in this temporal continuity by adsorbing, stabilizing, and slowly releasing reactive molecules, thereby extending their lifetimes and reducing their susceptibility to degradation. Such temporal coupling could have enabled both molecular preservation and functional integration, allowing natural selection to operate on increasingly stable and interactive molecular assemblies. Over time, this stepwise process could ultimately give rise to a complete evolutionary pathway toward life.

Furthermore, extraterrestrial inputs likely augmented Earth’s prebiotic inventory. Impacts by comets, meteorites, and interplanetary dust particles delivered organic precursors and catalysts (Fig. 2c), in some scenarios, potentially entire reaction environments through impact-generated hydrothermal systems [104,105]. While the panspermia hypothesis remains speculative, findings such as suspected microbial traces in Martian meteorites [106], highlight the possibility that life’s ingredients and perhaps some stages of prebiotic chemistry were not strictly confined to a single planetary setting.

Overall, the origin of life is more plausibly interpreted not as a localized event, but as a spatially distributed and temporally extended continuum. Mineral-mediated reactions operating in diverse geological environments, and linked by transport processes and environmental cycling, could have progressively increased chemical complexity on the early Earth. In this view, life emerged through a cascade of geochemically and mineralogically enabled steps that transformed simple molecules into increasingly complex and integrated prebiotic systems.

## 4 Mineralogical indicators for assessing planetary originability

### 4.1 Summarizing mineralogical roles in prebiotic synthesis

Based on current studies, here we summarize in Fig. 3 how major minerals may have supported different stages of chemical evolution. This highlights that owing largely to distinct physicochemical properties, different minerals are effective at different stages of prebiotic synthesis. Specifically, metal sulfides such as FeS and NiS, are effective in the synthesis of organic precursors and basic building blocks (e.g., carbon and nitrogen reduction, amino acid synthesis and peptide bond formation). As discussed before, these functions are commonly attributed to reactive surface sites provided by metal cations in sulfide minerals, which enable the adsorption, activation, and catalytic transformation of small molecules [2,12]. Notably, as discussed in Section 3.1, the reactions for synthesizing precursors often occur in high-energy environments, in which low-valence sulfur can supply sufficient reducing equivalents (electrons) to drive reduction reactions. In comparison, metal oxides and hydroxides mainly contribute to building blocks synthesis (e.g., amino acids and nucleobases), which requires lower energy inputs. In these cases, redox reactions can be readily driven by delocalized *d* electrons within transition metal–oxygen coordination polyhedrons [38,39]. Clay minerals, particularly montmorillonite, seem to be more effective at later stages, facilitating polymerization (e.g., peptide and oligonucleotide formation), vesicle assembly, and preserving reactive organic species. Their unique layered silicate structure allows organic monomers to be adsorbed and concentrated within interlayers, thereby promoting polymerization [72,75]. In addition, strong adsorption onto clay surfaces can stabilize molecular structures [80], while interlayer spaces may provide protection against radiation and other harsh environmental stresses.

**Fig. 3:**
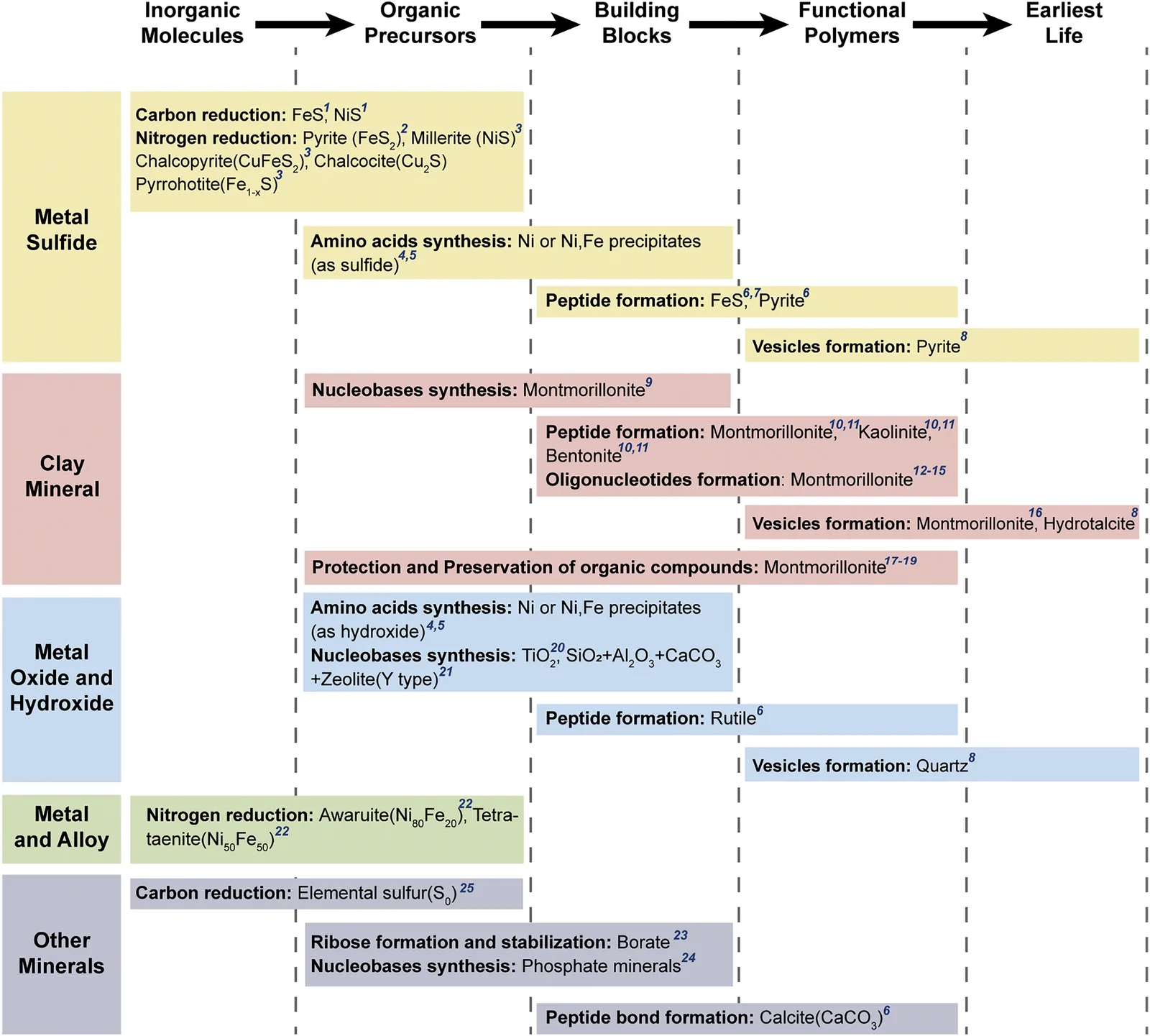
**Graphic summary of mineral roles documented in prebiotic synthesis.** Stages of chemical evolution adapted from Kitadai and Maruyama [22]; detailed mineral references are provided in Table S1 (Online).

It is noteworthy that the contributions of minerals in the origin of life are complicated and many aspects remain unknown. The summary presented in Fig. 3 is therefore not exhaustive, but rather a working framework based on currently available evidence. Many mineral functions in chemical evolution remain uncertain, and further experimental, theoretical, and geological studies will be needed to refine and expand this scheme. The mineral diversity during Hadean was much lower than today [6,7], and some elements essential for prebiotic synthesis may not have occurred as major mineral phases but rather as minor constituents within more abundant minerals. For example, much of the phosphorus in the oceanic crust may not have been present primarily as apatite, but instead incorporated as a trace component in mafic silicates (e.g., olivine) [107,108]. Therefore, it is necessary to consider the geochemical diversity and element availability when analyzing geological factors for the origin of life in order to obtain a comprehensive understanding.

### 4.2 Mineralogical assemblage indicators for astrobiological detection

A key pattern emerging from Section 4.1 is that no single mineral type appears to be actively involved in every stage of chemical evolution. Instead, different mineral groups seem to support different steps in prebiotic synthesis. This suggests that the co-occurrence of complementary mineral groups may be more informative than the presence of any individual mineral alone. If multiple mineral types occur within the same environment, or within spatially connected environments, they may collectively provide the catalytic, adsorptive, redox, and protective functions required across different stages of chemical evolution, thereby enabling a cascade of successive prebiotic reactions. On this basis, we propose mineralogical assemblage indicators to assess the potential for abiogenesis on rocky exoplanets. For example, according to Fig. 3, an environment containing both metal sulfide and metal oxides may support the formation of organic precursors, building blocks, and some functional polymers. If metal sulfides, metal oxides and clay minerals are all present, one may infer that the environment has the potential to support a broader range of prebiotic stages, possibly extending from small-molecule synthesis to polymerization and molecular preservation. By contrast, an environment containing only clay minerals might favor later-stage processes such as polymerization or stabilization, but may be less able to support the earlier synthesis of key precursors in the absence of more redox-active mineral phases. At present, the framework proposed here emphasizes assemblages of major mineral groups rather than individual species, and more specific candidate minerals can be identified from Fig. 3. Also, it should be noted that current knowledge of mineral-mediated prebiotic reactions is derived largely from isolated experiments and simulations, and the potential synergies or inhibitions arising from mineral assemblages in a specific setting remain poorly understood. Thus, these assemblage indicators should not be regarded as proof that certain minerals directly caused life to emerge, but rather as a theoretical tool for evaluating geological environments that may have been favorable for abiogenesis.

This framework is particularly relevant to astrobiology because it addresses a concept more specific than habitability alone. Generally, habitability refers to the capacity of a planetary body to support the activity or survival of living organisms [109]. However, a habitable environment does not necessarily possess the conditions required to generate life in the first place. The conditions required for abiogenesis are likely narrower, more restrictive, and still poorly constrained. To distinguish these concepts, it would be useful to introduce the notion of “originability”, defined here as the capacity of an environment to support the emergence of life from prebiotic chemistry. Therefore, the mineralogical framework developed here is intended to evaluate the geological originability of a planetary environment, rather than habitability.

In astrobiology, biosignatures are generally defined as signals generated directly or indirectly by biological activities. These biosignatures can be categorized into gaseous biosignatures (e.g., O_2_ [110], CH_4_ [111], PH_3_ [112]), surface biosignatures (e.g., the vegetation “red edge”, VRE [110]) and temporal biosignatures [23]. Despite their diversity, the rationale behind traditional biosignatures is to use remote sensing data to detect signs of extant or past biological activities, which are susceptible to both degradation and false-positive interpretation from abiotic processes, such as CH_4_ form by water-rock reactions [113] and VRE analogues by semiconductor minerals [114]. Technical challenges including spectral overlap, detection resolution, detection limit may lead to false negatives [23]. Moreover, because biological activity itself can be variable, some gaseous or temporal biosignatures may not be stable over long intervals.

Mineralogical assemblage indicators offer a complementary perspective. Unlike many gaseous or temporal biosignatures, mineralogical features can persist over planetary timescales. More importantly, they do not aim to detect life directly, but rather to identify environments where key prebiotic reactions may have been thermodynamically and kinetically feasible. As discussed before, these mineralogical assemblage indicators can evaluate the planetary potential to support certain stages of chemical evolution, and eventually the genesis of life. Therefore, integrating mineralogical assemblage indicators with traditional biosignature detection could improve the interpretation of astrobiological observations by linking biological potential to geological context. Furthermore, current research on habitable exoplanets typically focuses on the planet-scale atmospheric composition tectonics, and interior-surface interactions [115]. Mineralogical assemblage indicators provide a complementary and more localized geological framework, in which either local or regional mineral occurrences may indicate the potential for originability.

The application of this framework, however, depends on the ability to detect minerals beyond Earth, especially the accurate identification of poorly crystalline minerals. Extraterrestrial mineral identification so far relies on remote sensing data and spectral analysis. For example, minerals on Mars can be distinguished by their distinct spectral features. However, the confidence of results may be affected by atmospheric aerosol interference, surface-obscuring dust, overlapping spectral features, and the inherent limitations of spectral models [23,116]. Nevertheless, recent study has shown progress in quantitative identification of poorly crystalline Mn minerals on Mars by integrating a deep learning framework in spectral interpretation [117]. Moreover, recent advances in space telescopes and observational facilities shed light on the future application of this mineralogical framework. Detailed observation and distinction of specific mineralogical signals on rocky exoplanets may become possible by using the mid-infrared instrument on James Webb Space Telescope (JWST) [118]. Future missions and observatories such as LUVOIR and HabEx may further expand the feasibility of applying this framework to the detection of exoplanetary environments with high geological originability [119,120].

## 5 Conclusion

The origin of life was likely a multistage process spanning the synthesis of prebiotic precursors, the assembly of functional polymers, and the emergence of protocells. Minerals likely provided indispensable contributions at each step: transition-metal sulfides, oxides, and related minerals catalyzed the formation of precursors and building blocks, whereas clay minerals promoted polymerization, stabilization, and compartmentalization. These processes were unlikely to have occurred in a single environment, but rather across diverse geological settings linked through spatial and temporal coupling. Recognizing this distributed and mineral-mediated cascade is essential for reconstructing plausible prebiotic pathways. In astrobiology, this review suggests that mineralogical assemblages on rocky exoplanets may serve as stable indicators of prebiotic potential and geological originability. Combined with traditional biosignature approaches and technological breakthroughs, such indicators may improve our ability to identify planetary environments with originability.

## CRediT authorship contribution statement

**Houze Lu:** Writing – review & editing, Writing – original draft, Methodology, Investigation, Formal analysis, Conceptualization. **Yan Li:** Writing – review & editing, Writing – original draft, Supervision, Methodology, Conceptualization. **Dehai Liang:** Writing – review & editing.

## Declaration of competing interest

The authors declare that they have no conflicts of interest in this work.

## Appendix Supplementary materials

Image, application 1
